# Effect of duration of fluoride exposure on the reproductive system in male rabbits

**DOI:** 10.4103/0974-1208.74159

**Published:** 2010

**Authors:** Naresh Kumar, Sushma Sood, B Arora, Manjeet Singh, Beena

**Affiliations:** Department of Physiology, Pt B.D. Sharma Post Graduate Institute of Medical Sciences, Rohtak, Haryana, India; 1 Department of Pathology, Pt B.D. Sharma Post Graduate Institute of Medical Sciences, Rohtak, Haryana, India

**Keywords:** Fluoride, sperm count, testis

## Abstract

**BACKGROUND AND OBJECTIVES:**

Fluorosis has become an endemic problem worldwide. Fluoride has its effect on various organs, including the reproductive system, although there are controversial reports over it. Thus, the present study was designed to study the effect of sodium fluoride (NaF) exposure for different durations.

**MATERIALS AND METHODS:**

30 healthy rabbits were divided into three equal groups. Group I was fed on a standard diet for 30 days, Group II was fed on sodium fluoride (20 mg/kg body weight) for 30 days and Group III was fed on sodium fluoride (20 mg/kg body weight) for 60 days. Sperm count, motility, progressive motility and weight of testis and epididymis were measured and compared with the control group.

**RESULTS:**

We observed that in the group exposed to NaF for 30 days (Group II), there was a significant decrease in all the parameters except the testicular weight and in the group on exposure of 60 days (Group III), there was a significant decrease in all the parameters when compared with the control group (Group I). On applying the Tukey *post-hoc* test between Groups II and III, we observed that there was a significant decrease in the sperm count while the other parameters showed a non-significant decrease. Testicular histology was confirmatory for the above findings.

**CONCLUSION:**

The present study demonstrates that fluoride hampers the reproductive functions of male rabbits and is proportional to the duration of fluoride exposure.

## INTRODUCTION

Humans are exposed to sodium fluoride (NaF) from a number of sources, including water, medicines, pesticides, insecticides, fertilizer residues, dental restorative materials, dental products (tooth pastes and mouth rinses), pediatric supplements, beverages prepared with fluoridated water and food.[1] Fluorosis is an endemic public health problem in nearly 22 nations around the world. The World Health Organization (WHO) guideline is that 1.5 ppm of fluoride is the desirable upper limit in drinking water. The magnitude of problem of fluorosis in India is roughly estimated to be of 66.62 million people at risk.[2] The recommended levels of fluoride in drinking water are 0.5–0.8 mg/L. Fluoride levels above 1.5 mg/L may lead to dental fluorosis while levels above 3–6 mg/L during the life time may lead to skeletal fluorosis.[3] Involvement of the reproductive organs due to fluorosis in animals had also been studied extensively. Messar *et al*., reported that the low levels of fluoride in food rendered mice infertile, while a high-fluoride diet improved their fertility.[3] Chinoy and Sequeira reported that sodium fluoride treatment in mice caused an alteration in the histology of reproductive organs and morphology of sperm and induced biochemical changes.[4] These reports were contradicted by Tao and Suttie, whose experiments showed that fluoride did not play any essential role in reproduction.[5] Because of these controversial reports, we intended to assess the effect of fluoride on the rabbit reproductive system.

## MATERIALS AND METHODS

The study was conducted in the Department of Physiology in collaboration with the Department of Pathology after approval by the Institutional Animal Ethical Committee. Thirty healthy, adult New Zealand white male rabbits were used for the study. All the rabbits were of the same age group, with a weight range of 1.5–2.5 kg. The rabbits were housed in a well-ventilated animal house and caged separately, at a temperature of 29-32°C and exposed to 10–12 h of daylight. They received food and water *ad libitum*. Sodium fluoride (Ranbaxy Laboratories, Chandigarh, India) was given using a feeding tube attached to a hypodermic needle in the dose of 20 mg/kg body weight/day.[6] The rabbits were divided into three equal groups of 10 each.

- Group I: (Untreated control) The rabbits were maintained on a standard diet and water *ad libitum* for 30 days and were sacrificed on the 31^st^ day.
- Group II: The rabbits were given sodium fluoride (NaF) for 30 days and were sacrificed on the 31^st^ day.
- Group III: The rabbits were given sodium fluoride (NaF) for 60 days and were sacrificed on the 61^st^ day.

The control and the test group rabbits were anesthetized with intravenous injection of Urethane (0.5–1.5 g/kg of body weight). Incision was given on the scrotum of the rabbit and the testis and the epididymis were carefully exposed, removed and were subjected to the following physiological and histopathological studies:

### Epididymal sperm count

From each separated epididymis, the cauda part was removed and placed in a beaker containing 1 mL physiological saline solution. Each section was quickly macerated with a pair of sharp scissors and left for a few minutes to liberate its spermatozoa into the saline solution. Sperm count was performed under the microscope using the improved Neubauer chamber (Fein-Optik, Blankenburg, Germany).[7]

### Sperm motility and progressive sperm motility

A drop of semen was placed on the slide and two drops of warm 2.9% sodium citrate was added. The slide was covered with a cover slip and examined under the microscope using ×40 objective for sperm motility. Sperm motility and progressive motility were determined.

### Weight of the testis and the epididymis

The weight of the testis and the epididymis of all the groups was taken.

### Histopathology of the testis

The testis was removed after being freed from the surrounding tissue. The tissue was kept in a 10% neutral formalin solution for fixation. After 1 week, the tissue was washed for 24 h under running tap water, then dehydrated through ascending grades of alcohol, cleared in xylene and embedded and blocked in paraffin. Sections of 4–5-µm thickness were taken and stained with hematoxylin and eosin and were examined under the microscope.

### Statistical analysis

The values of different parameters like sperm count, sperm motility, progressive motility and weight of the testis and epididymis were compared using one way ANOVA with *post-hoc* Tukey test and the statistical values were expressed as mean ± SD [Table 1].

**Table 1:** Comparison of epididymal sperm count, motility and progressive motility of sperm, weights of testis and epididymis among Group I (control), Group II (fed on NaF for 30 days) and Group III (fed on NaF for 60 days) using ANOVA and *post-hoc* Tukey test

Parameters	Group I Mean ± SD	Group II Mean ± SD	Group III Mean ± SD	ANOVA “*F*”-value	*P*-value
Sperm count × 10^6^/epididymis	162 ± 10.52	111.75 ± 17.48*	91.5 ± 11.25†,‡	72.777	0.000
Sperm motility (%)	72.1 ± 3.28	59.1 ± 10.28*	55.9 ± 7.24†	13.053	0.000
Progressive motility of sperm (%)	58.6 ± 6.02	47.1 ± 11.44*	39 ± 7.02†	13.436	0.000
Testicular weight (g)	3.22 ± 0.46	2.86 ± 0.37*	2.63 ± 0.42†	4.931	0.015
Epididymal weight (g)	0.63 ± 0.21	0.46 ± 0.06*	0.45 ± 0.05†	5.835	0.008

* *Post-hoc* Tukey test (*P* < 0.05) comparison between Groups I and II;

† *Post-hoc* Tukey test (*P* < 0.05) comparison between Groups I and III;

‡ *Post-hoc* Tukey test (*P* < 0.05) comparison between Groups II and III

## RESULTS

On comparing the three groups using one way ANOVA, there was a statistically significant difference among the three groups [Table 1]. On multiple comparisons using Tukey’s *post-hoc* test, we observed that in the group exposed to NaF for 30 days (Group II), there was a significant decrease in all the parameters except the testicular weight and in the group on exposure of 60 days (Group III), there was a significant decrease in all the parameters when compared with the control group (Group I). On applying the Tukey *post-hoc* test between Groups II and III, we observed that there was a significant decrease in the sperm count while the other parameters showed a non-significant decrease.

### Histopathology of the testis

**Figure 1:**
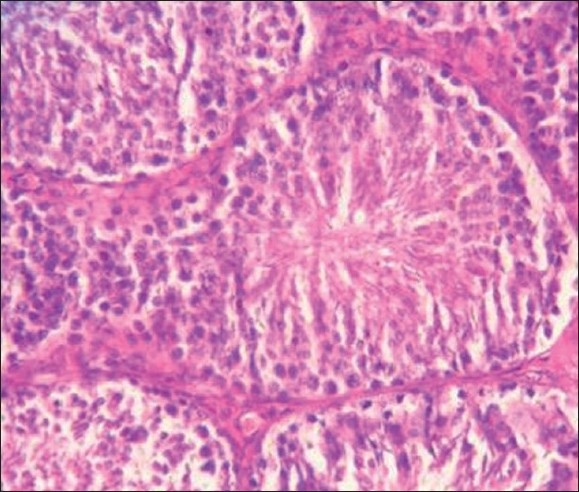
Histopathology of the testis in rabbits fed on a standard diet

Group I [Figure 1]: Histology of the testis showed normal spermatogenesis with different stages of differentiation and maturation. The spermatozoa were in groups attached to the inner aspect of the lumen of the seminiferous tubules.

**Figure 2:**
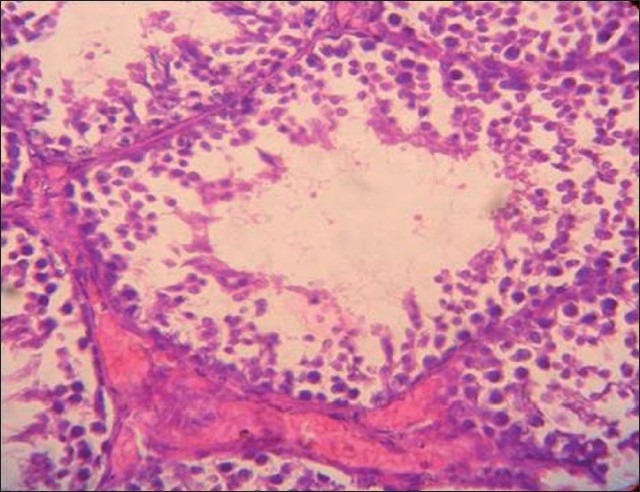
Histopathology of the testis in rabbits fed on NaF (20 mg/ kg) for 30 days

Group II [Figure 2]: There was a lack of differentiation and maturation of spermatocytes and there was marked infiltration in the interstitial area of the seminiferous tubules. No mature spermatozoa were seen in the lumens of the seminiferous tubules.

**Figure 3:**
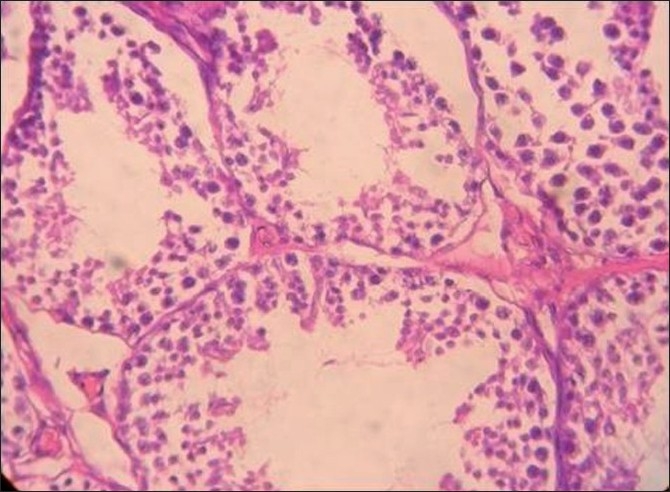
Histopathology of the testis in rabbits fed on NaF (20 mg/ kg) for 60 days

Group III [Figure 3]: There was a marked atrophy and necrosis of the seminiferous tubules and no normal spermatocytes or spermatids were seen. There was complete cessation of spermatogenesis and the seminiferous tubules were devoid of spermatozoa. This group also showed that there was sloughing off of the spermeogenic cells in the luminal region of the seminiferous tubules of the testis, leading to disorganization of their epithelium.

## DISCUSSION

Animal studies had shown contradictory reports regarding the effect of fluorosis on the reproductive system. Therefore, the present study was performed to establish the link between fluoride and its effect on sperm count, motility, progressive motility, weight of testis, weight of epididymis and histopathology of the testis of age and body weight matched groups.[6]

### Effect on sperm count

It was observed that in comparison with the control group, there was a significant decrease in the epididymal sperm count when NaF was given for 30 days to rabbits and a greater decrease when NaF was given for 60 days. Hence, the present study proved that higher the fluoride exposure, higher the effect on sperm counts. Similar results had been observed in rats, mice and rabbits in studies performed earlier.[8–10]

The effect of fluoride toxicity on spermatogenesis could be because fluoride reduces the testosterone levels and by reducing the testicular zinc levels, it impairs angiotensin-converting enzyme (ACE) activity and hence causes inhibition of spermatogenesis.[1] Apart from the direct effect on the levels of testosterone, fluoride inhibits androgen receptor (AR) mRNA expression in Sertoli cells and causes a decrease in AR through which testosterone acts.[11] Wan *et al*., reported that epidermal growth factor (EGF) and its receptor (EGFR), which plays an important role in male reproductive functions of rat, were significantly decreased in Leydig cells, spermatogonia and spermatocytes on exposure to 68 ppm fluoride for 10 days.[12] Other factors responsible for the arrest of spermatogenesis might be the lack of available proteins necessary for cell division, growth and differentiation of germ cells. In a study, disruption of normal cell cycle and apoptosis was indicated at doses of 200–30 mg/L of fluoride, which may be attributed to the blockage of the G1 phase of the cell cycle. Another mechanism of apoptosis is increased levels of oxidants, which damages the DNA.[13]

### Effect on motility

In the present study, there was a significant decrease in sperm motility and progressive motility in the group fed with fluoride 20 mg/kg for 30 days and 60 days as compared with the control. Huang *et al*., also reported a significant reduction in the sperm motility of mice fed on 100, 200 and 300 mg NaF/L for 8 weeks as compared with the control group.[13] Similar results were shown in rats and mice in many other studies.[9,14–16] One important study demonstrated that human spermatozoa lost their motility *in vitro* in the presence of 250 mM fluoride within 20 mins of exposure and, similarly, fluoride (30 mM) made the bull sperms immotile within 2 min at 30°C *in vitro*.[8]

The mechanism by which fluoride affects sperm motility has not been clearly elucidated. However, it has been postulated that fluoride could act directly on the motile apparatus without affecting other metabolic systems.[17] One mechanism could be decline in the fructose level, which provides energy for motility, in the seminal vesicle and vas deferens due to alteration in carbohydrate.[8,17] Fluoride may also act by inhibiting many enzymes: the first mode of action is that fluoride binds with cofactors like Mg, Ca, Zn and Se and thus inhibits glycolysis, respiration and motility of sperms, or it might also form insoluble complexes with magnesium or phosphates in enzymes like enolase and acid and alkaline phosphatase.[17] Another reason for decreased sperm motility was decreased level of androgen carrier proteins involved in sperm motility.[18] Furthermore, structural defects were observed in the flagellum, acrosome and nucleus of spermatids and epididymal spermatozoa of fluoride-treated rabbits (10 mg/kg/day for 18 months) leading to abnormal motility.[19]

### Organ weights

In our study, there was a significant decrease in the epididymal weight in Groups II and III as compared with the control while there was a significant decrease in the testicular weight in Group III only, indicating that a higher level of NaF is needed to affect testicular weight than epididymal weight. In other words, we can conclude that fluoride affects epididymal weight earlier than testicular weight.

Similarly, in another study, rabbits fed on fluoride were having a significant decrease in epididymal weight.[20] Also, the weight of the cauda epididymis in fluoride-treated (10 mg/kg for 30 days) mice declined significantly compared with the control groups.[9] However, no significant difference was observed in the mean testicular weights of rats fed on 100 and 300 ppm fluoride for 12 weeks when compared with the control. It might be because of the lower fluoride concentration.[16] Our findings differed from those of Ghosh *et al*., who reported an increase in the relative testis weight of rats as compared with the control with fluoride treatment (20 mg/kg for 29 days), which might had been due to a compensatory change or due to fluid accumulation in the testis.[21]

### Testicular histology

Figures 1–3 show that longer the fluoride exposure, higher was the damage to the reproductive system.

There was a lack of differentiation and maturation of spermatocytes, with no mature spermatozoa seen in the lumens in Group II [Figure 2] and complete cessation of spermatogenesis in Group III [Figure 3] as compared with normal spermatogenesis in the control group [Figure 1]. Thus, our results were similar to a study that showed that 30 days of treatment with sodium fluoride (10 mg/kg body weight) to mice resulted in sloughing off of the spermatogenic cells in the luminal regions of the seminiferous tubules of the testis, leading to disorganization of their epithelium, which caused a complete absence of spermatogenesis in the testis.[18] Also, direct injection of sodium fluoride (50 µg/50 µl) into the vas deferens resulted in spermatogenic arrest, the absence of spermatozoa in the seminiferous tubules, a decreased sperm count in the cauda epididymis and a decrease in fertility,[22] while Sprando *et al*., showed that rats fed on a 250 ppm fluoride for 10 weeks showed no distinguishable change in testicular histology from their control group.[1] This might be due to a lower level of fluoride. Susheela *et al*., in their study on rabbits found that there was disruption of spermatogenic cells in the seminiferous tubules that were degenerated and devoid of spermatozoa. In animals treated for 18 or 29 months, loss of cilia on the epithelial cells lining the lumen of the ductuli efferentes of the caput epididymidis and of the stereocilia on the epithelial cells lining the lumen of the vas deferens was also observed. Similarly, in our study, there was a lack of spermatogenesis, as evident on testicular histology.[10]

## CONCLUSION

Fluoride definitely has adverse effects on the reproductive functions of male rabbits, and these effects are more pronounced with the increasing duration of exposure.
